# Capitalizing on facilitators and addressing barriers when implementing active tuberculosis case-finding in six districts of Ho Chi Minh City, Vietnam: a qualitative study with key stakeholders

**DOI:** 10.1186/s13012-021-01124-0

**Published:** 2021-05-19

**Authors:** Olivia Biermann, Phuong Bich Tran, Rachel Jeanette Forse, Luan Nguyen Quang Vo, Andrew James Codlin, Kerri Viney, Maxine Caws, Knut Lönnroth

**Affiliations:** 1 Department of Global Public Health, Karolinska Institutet, Stockholm, Sweden; 2 Faculty of Medicine and Health Sciences, University of Antwerp, Antwerp, Belgium; 3 Friends for International Tuberculosis Relief, Ho Chi Minh City, Vietnam; 4 Research School of Population Health, College of Health and Medicine, Australian National University, Canberra, Australia; 5 Department of Clinical Sciences, Liverpool School of Tropical Medicine, Liverpool, UK; 6 Birat Nepal Medical Trust, Lazimpat, Kathmandu, Nepal

**Keywords:** Tuberculosis, Facilitators and barriers, Active case-finding, Community-based screening, Patients, Volunteers, Employees, Leaders, Vietnam, Qualitative research

## Abstract

**Background:**

Vietnam has a high burden of undetected tuberculosis (TB). The Vietnamese National TB Strategic Plan highlights active case-finding (ACF) as one strategy to find people with TB who are currently unreached by the existing government health services. The IMPACT TB (Implementing proven community-based active TB case-finding intervention) project was implemented across six districts of Ho Chi Minh City, 2017–2019. We aimed to explore the facilitators and barriers for ACF implementation during the IMPACT TB project to understand how and why the intervention achieved high yields.

**Methods:**

This was an exploratory qualitative study based on 39 semi-structured key-informant interviews with TB patients who were diagnosed through ACF, employees and volunteers who implemented ACF, and leaders from district, national, or international institutions and organizations in Vietnam. Thematic analysis was applied, using an implementation science framework by Grol and Wensing.

**Results:**

We generated three main themes: (1) the studied ACF model used in Vietnam provided a conducive social and organizational context for ACF implementation with areas for improvement, including communication and awareness-raising, preparation and logistics, data systems and processes, and incentives; (2) employees and volunteers capitalized on their strengths to facilitate ACF implementation, e.g., experience, skills, and communication; and (3) employees and volunteers were in a position to address patient-level barriers to ACF implementation, e.g., stigma, discrimination, and mistrust. These themes covered a variety of facilitators and barriers, which we divided into 17 categories. All categories were mentioned by employees and volunteers, except the category of having a network that facilitates ACF implementation, which was only mentioned by volunteers. This study also highlighted examples and ideas of how to address facilitators and barriers.

**Conclusions:**

IMPACT TB provided a favorable social and organizational context for ACF implementation. Individual employees and volunteers still determined the success of the project, as they had to be able to capitalize on their own strengths and address patient-level barriers. Volunteers especially used their networks to facilitate ACF. Knowledge of both facilitators and barriers, and how to address them can inform the planning and implementation ACF in Vietnam and similar contexts across low- and middle-income countries worldwide.

**Supplementary Information:**

The online version contains supplementary material available at 10.1186/s13012-021-01124-0.

Contributions to the literature

- We provide insights into facilitators, barriers, and “how-to” strategies for active case-finding (ACF) implementation in Vietnam, applying an implementation science framework.
- We captured perceptions from key stakeholders involved in ACF implementation, including patients, employees, volunteers, and leaders.
- Employees and volunteers experienced similar facilitators and barriers for ACF. A single facilitator was mentioned by only volunteers: the use of one’s network to facilitate ACF.
- Despite a conducive social and organizational context for ACF implementation, employees and volunteers still make or break the process because addressing facilitators and barriers for implementation depends on their individual skills and efforts.

## Background

Annually, approximately 10 million people fall ill with tuberculosis (TB), of whom 2.9 million people are not notified as having been diagnosed and treated [1]. The World Health Organization’s (WHO) End TB Strategy highlights active case-finding (ACF) as one strategy to find the people with TB who are currently being missed by health services [2]. ACF is defined by WHO as the “systematic identification of people with suspected active TB, in a predetermined target group, using tests, examinations or other procedures that can be applied rapidly” [3]. It is synonymous with systematic screening for active TB, while it implies screening outside of health facilities [3]. ACF has been shown to find more people with TB at an earlier stage of disease [4, 5] compared to “passive case-finding” (PCF). PCF is the standard approach to TB case-finding, relying on people seeking care when they have signs and symptoms of TB [6]. ACF ideally supplements rather than replaces PCF. As for any other screening intervention, the potential benefits and harms of ACF must be assessed in any given context [3, 7].

Thirty low- and middle-income countries, among them Vietnam, account for almost 90% of the global TB burden [8]. In Vietnam, the estimated prevalence of TB among adults was 322 per 100,000 population, based on a recent national TB prevalence survey [9]. In 2019, the estimated gap between incident and notified TB cases in Vietnam was 65,495 (39%) [10]. Contributing factors to the TB detection gap in Vietnam are limited awareness of TB, lack of access to health care [11], and stigma [12]. Aligned with WHO guidelines, the Vietnamese National TB Strategic Plan emphasizes the role of ACF for TB elimination [13], while the government passed legislation to end TB by 2030 [14]. In the past decade, different ACF models have been implemented in the country [13]. A cluster randomized controlled trial found declines in TB burden through systematic implementation of active community-wide screening in rural Vietnam [15].

While ACF should be appropriately coordinated and integrated within the health system [3, 16], it is often described as limited by human and financial resources [17, 18]. A recent scoping review summarized the facilitators and barriers for ACF implementation [19]. Factors related to implementers included experience, skills, and motivation [20–22]. However, based on the aforementioned review, there was little evidence on how to address facilitators and barriers [19]. To the best of our knowledge, evidence on the facilitators and barriers for implementing ACF in Vietnam, including “how-to” strategies, is lacking.

IMPACT TB (Implementing proven community-based active TB case finding intervention; www.impacttbproject.org) was a Horizon 2020-funded project on ACF in Nepal and Vietnam. In Vietnam, the project was implemented in Ho Chi Minh City (HCMC) between October 2017 and September 2019. The ACF model applied during IMPACT TB generated a substantial yield of TB cases (354 per 100,000 population), while TB notifications in the project’s intervention area increased by 12.3% [23]. Most indicators along the TB care cascade were similar between employees and volunteers who implemented ACF. The Number Needed to Screen to detect one case of TB (calculated by dividing the number of persons screened by the number of persons diagnosed with TB) was 2.7 times higher for employees compared to volunteers. Yet, changes in TB treatment rates were higher in the employee districts, particularly for patients with bacteriologically confirmed TB [23]. A knowledge gap remains in terms of identifying the facilitators and barriers for implementing ACF and shedding light on how these factors had been addressed, which is the focus of this study.

## Methods

### Aim and design

This was an exploratory qualitative study based on semi-structured key-informant interviews [24]. We reported the study using the COREQ (Consolidated criteria for reporting qualitative research) Checklist [25] (Additional file 1).

The study explored the facilitators and barriers linked to IMPACT TB ACF implementation in six districts of HCMC. Our research question was: How do key stakeholders perceive the facilitators and barriers for the implementation of IMPACT TB ACF in six districts of HCMC?

### Setting of the study and the “implementation object”

The setting of the study and the ACF intervention as the “implementation object” have been previously described in detail [23]; however, we provide a brief overview here. Salaried employees implemented ACF in the HCMC districts of *Hoc Mon*, *Tan Binh*, and *District 12*, while incentivized volunteers implemented the same ACF interventions in *Binh Chanh*, *Districts 6* and *8* (from here on referred to as “employees” and “volunteers”). The eligibility and selection criteria for both employees and volunteers were identical, e.g., in terms of education and age. These staff were identified and recruited by district authorities among retired health staff, civil society members, community volunteers, and former TB patients who had been living in the district for at least five years [23]. The sole distinction lay in the remuneration, i.e., employees received a salary of 136 USD/month, while volunteers received a stipend of 23 USD/month. All employees and volunteers had the same responsibilities within the project, participated in capacity-building activities and received performance-based incentives [23].

The employees and volunteers screened household and other close contacts of index TB cases (TB patients notified by the District TB Units). ACF also included persons from high-risk groups, such as neighbors of a TB patient [26], people living in slums, and boarding homes. All participants had verbal screens for symptoms and history of TB, which were recorded into a custom-built app installed on a tablet [23].

Household contacts were referred for a chest X-ray (CXR) irrespective of whether they had TB symptoms due to the high rate of asymptomatic TB patients in Vietnam [9, 27]. All other persons (without confirmed household contact) were only referred for CXR if they reported any symptom suggestive of TB. People referred for CXR received a transport voucher and a free CXR at the District TB Unit or community screening events held during weekends. Those with an abnormal CXR were then tested using the Xpert MTB/RIF® assay. Sputum smear microscopy was used for those who had a normal CXR but any symptoms suggestive of TB, and those who did not get a CXR. Employees and volunteers transported sputum samples for people who were unable to reach the laboratories. Symptomatic persons with negative sputum test results underwent clinical evaluation by the District TB Unit and the Pham Ngoc Thach Hospital, in line with the national policy. If diagnosed with TB, employees and volunteers assisted patients with drug-susceptible TB to access TB treatment, including support to provide proof of residency, if needed, and patient counseling and psychosocial support. Site coordinators assisted the District TB Units with data collection and reporting. Patients with drug-resistant TB were referred to Pham Ngoc Thach Hospital for further evaluation and treatment [23].

### Characteristics of the interviewees, recruitment, and sample selection

Interviewees were purposively sampled and included three different stakeholder groups: (1) TB patients identified through ACF, (2) employees and volunteers implementing ACF, and (3) leaders. Leaders included District TB Unit leaders from the six districts; representatives of the National TB Programme, of the Centre for Community Development and of international organizations based in Vietnam, namely the United States Centers for Disease Control and Prevention, the Clinton Health Access Initiative, the WHO, the International Union Against TB and Lung Disease, and the Woolcock Institute of Medical Research.

The research team compiled an initial list of the types of interviewees to be included in the study. IMPACT TB coordinators in the different districts suggested volunteers and employees to be interviewed (stakeholder group 1). We later triangulated the selection of volunteers and employees with their performance data, which revealed that the selected volunteers were among the top 25% and the employees among the top 50% in terms of key performance indicators. The employees and volunteers suggested TB patients (stakeholder group 2) who had to be on TB treatment or have completed it. LNQV (co-author) suggested interviewees for stakeholder group 3.

A local research assistant (Linh Hoang, LH) contacted volunteers, employees, and TB patients to explore their interest in participating in the study. Co-author PBT contacted potential interviews for stakeholder group 3. If the potential interviewees were interested, LH and PBT set up interview appointments. Overall, 41 interviewees were contacted; 39 (95%) agreed to participate. Two persons (stakeholder group 3) declined participation due to lack of time or interest. Table 1 provides an overview of the interviewees.

**Table 1:** Overview and characteristics of the interviewees

Stakeholder groups	*n*	Number and proportion of female participants
Employees and volunteers (stakeholder group 1)	12	10 (83%)
Incentivized volunteers	6	6 (100%)
Salaried employees	6	4 (67%)
Patients (stakeholder group 2)	12	4 (33%)
Leaders of stakeholder institutions (stakeholder group 3)	15	4 (27%)
District level	6	1 (17%)
National level	3	0 (0%)
International level	6	3 (50%)
Total	39	18 (46%)

### Data collection

Lead author OB developed interview guides with open-ended questions for each stakeholder group (Additional file 2) which co-authors RJF, MC, KL, and KV provided feedback on. The interview guides for stakeholder groups 1 and 2 contained the same content with language adjustments to ensure clarity. The questions were designed to ask what interviewees liked or found challenging about the implementation of the IMPACT TB ACF activities, aspects they thought could be improved, and how. The interview guide for stakeholder group 3 comprised an additional section about ACF data. The interview guides were translated into Vietnamese and back translated into English. Being bi-lingual, PBT ensured the quality of the translations.

Data were collected between October and December 2019. PBT and LH conducted the 39 semi-structured key-informant interviews. Each interviewee was given/read information about the study, provided written informed consent, and received a stipend of 300,000 VND (approx. 13 USD) for their participation and time. The first interviews were conducted as pilot interviews (two with each stakeholder group) after which the interview guides were revised, e.g., for stakeholder groups 1 and 2, questions were added on people refusing ACF participation. OB conducted the first interview in stakeholder group 3, PBT interviewed all remaining interviewees of stakeholder group 3, while LH conducted all interviews with stakeholder groups 1 and 2. Five interviews in stakeholder group 3 were in English, while all other interviews were conducted in Vietnamese. The 36 face-to-face interviews were conducted at interviewees’ homes, workplaces, international conferences, health stations, or District TB Units, depending on interviewees’ preferences. Three interviews (stakeholder group 3) were conducted via telephone.

During the majority of the interviews, only LH or PBT and the respective interviewee were present. The exceptions were the above-mentioned first interview in stakeholder group 3, during which both OB and PBT were present, and one interview during which the wife of the TB patient was present. The average duration of an interview was 31 min. The audio-recorded interviews were transcribed verbatim and were then translated into English by a professional translator. The anonymity and confidentiality of the interviewees were ensured by coding them as numbers and removing identifiers. The interviews were conducted beyond reaching information power to capture opinions from the diverse range of stakeholders [28].

### Data analysis

We conducted a data-driven thematic analysis [29] in *NVivo 11*, employing a realist approach considering the whole data set and reporting experiences, meaning and the reality of interviewees. Choosing a realist approach meant that we focused on the manifest rather than the latent content of the interviews [29]. OB and PBT jointly analyzed the data. First, OB coded five interviews. She discussed and revised the codes with PBT, and then regularly revised them for the remainder of the analysis. Second, a list of codes was developed that reflected the descriptions and ideas about ACF implementation. Codes were checked across the data set. Third, the authors used a framework by Grol and Wensing [30] entitled “Barriers to and incentives for change at different levels of healthcare” to review and structure the data. The framework describes how barriers and facilitators can be identified, categorized, and used for the development of a tailored intervention strategy. It categorizes facilitators and barriers into levels: the innovation (in our case, for ACF), the individual professional, the patient, the social context, the organizational context, and the external environment (e.g., political factors). We did not describe the external environment in our analysis to keep the article’s scope strongly focused on local implementation rather than policy issues. Fourth, the categories were created from observed patterns of meaning in the data and the theoretical understanding gained during the previous step. Fifth, the findings were mapped onto the framework to show their contributions to it, and to compare and contrast perceptions across the different groups of interviewees. Finally, three themes were identified that captured the meaning and association between the categories. The themes are presented in the results section. Table 2 provides an example of the coding process.

**Table 2:** Example of the coding process

Interviewee	Quote	Code	Category	Framework level	Theme
Interviewee #30, employee	“I come to meet them [people with presumptive TB], but they don’t believe me, so they tell me to go away. There’s so many con men, so people don’t want to believe. But if I keep trying to convince them, they will finally understand.”	Mistrust	Trust and mistrust	Patient	Employees and volunteers were in the position to address patient-level barriers to ACF implementation, e.g., stigma, discrimination and mistrust.

## Results

We generated three main themes from the data: (1) IMPACT TB provided a conducive social and organizational context for ACF implementation with areas for improvement, including communication and awareness-raising, preparation and logistics, data systems and processes, and incentives. (2) Employees and volunteers capitalized on their strengths to facilitate ACF implementation, e.g., experience, skills, and communication. (3) Employees and volunteers were in a position to address patient-level barriers to ACF implementation, e.g., stigma, discrimination, and mistrust. These themes showed that people are at the centre of ACF implementation. The themes cut across 17 categories, which we structured based on Grol and Wensing’s framework levels (Table 3). In the following section, we provide a narrative which summarizes the categories per framework level and provides examples of how to build on facilitators and overcome barriers.

**Table 3:** The 17 categories divided by framework level and specified as facilitator/barrier, and examples of how to build on facilitators and overcome barriers

Level		Categories	F	B	Examples of how to build on facilitators and overcome barriers	Highlighted in interview with			
						Patient	Employee	Volunteer	Leader
Innovation	1	Access to healthcare through ACF	x		IMPACT TB increased accessibility through mobile CXR events outside of working hours and during the weekend, and provided monetary incentives for ACF participants	X	X	X	X
	2	Free screening, testing and treatment	x		IMPACT TB offered free screening, transport vouchers to those referred for CXR, free CXR and support in enrolling for free treatment	X	X	X	X
	3	Support for TB patients	x		IMPACT TB provided financial support for food and fuel, besides free screening, testing and treatment	X	X	X	X
Individual professional	1	Dedication and motivation	x		Employees and volunteers adapted their schedules based on patients’ availability, and encouraged patients	X	X	X	X
	2	Experience and skills	x		Employees and volunteers used communication, persuasion and advising skills, and shared experience with each other	X	X	X	X
	3	Having a network that facilitates ACF implementation	x		Volunteers used their networks and relationships to easily find their way around in the districts and to approach people with suspected TB	X	-	X	-
	4	Good communication	x		Employees and volunteers spoke local dialects, knew local context, used tools such as flyers and communication channels such as social networking app *Zalo*	X	X	X	X
Patient	1	Limited participation		x	Employees and volunteers addressed stigma, discrimination, fear and mistrust (see next row)	X	X	X	X
	2	Prevalence of stigma, discrimination and fear		x	Employees and volunteers contacted patients on the telephone instead of visiting them at home, and invited whole communities instead of selected groups for ACF	X	X	X	X
	3	Trust and mistrust	x	x	Employees and volunteers communicated clearly and truthfully, were friendly and kind	X	X	X	X
Social context	1	Collaboration and engagement	x	x	IMPACT TB and employees and volunteers collaborated with and engaged District TB Units, health stations, committees, local leaders, residential groups, etc. Communication and awareness-raising should improve to further strengthen collaboration and engagement (see next row)	X	X	X	X
	2	Need to improve communication and awareness-raising		x	IMPACT TB should use a variety of communication channels including television, radio and online media, and made community-wide announcements; employees and volunteers should communicate directly with patients and visit individual households	X	X	X	X
	3	Commitment and support	x		IMPACT TB and employees and volunteers ensured all stakeholder had a common understanding of ACF	X	X	X	X
Organizational context	1	Availability of and need to retain human and financial resources	x		IMPACT TB offered human and financial resources for the project period (2017-2019)	X	X	X	X
	2	Importance and appreciation of capacity-building	x		IMPACT TB included capacity-building for employees and volunteers	-	X	X	X
	3	Incentives for employees and volunteers	x	x	IMPACT TB provided incentives; an increase of incentives should be considered	X	X	X	X
	4	Challenges with preparation and logistics		x	Employees and volunteers collaborated closely with District TB Units; IMPACT TB should prepare early, formulate clear implementation plan, and provide detailed instructions for employees and volunteers and District TB Units	X	X	X	X

*ACF* active case-finding, *IMPACT TB* Implementing proven community-based active TB case finding intervention, *CXR* chest X-ray, *TB* tuberculosis, *F* facilitator, *B* barrier

### Innovation

Interviewees from all stakeholder groups stressed the benefits of ACF. We identified three categories of benefits: access to healthcare, support for TB patients, and the availability of free screening, testing and treatment.

In terms of access to health care, interviewees elaborated on the benefits of mobile CXR; ACF participants could avoid traveling long distances, saved money and time, and received a monetary incentive for CXR testing. Moreover, accessibility was increased when mobile X-ray events took place outside of working hours and on weekends. Employees and volunteers also benefitted from accessible services, e.g., in terms of logistics.

In this program, I spent no money, and I was offered money for fuel and free medicines, it was in a nearby place and took little time. It’s not just me, everyone likes those things. (interviewee #13, patient)

Free screening, testing, and treatment were strong factors motivating participation in ACF, especially among people from underprivileged households. Moreover, being able to offer free services helped employees and volunteers in persuading people to participate in ACF.

People in difficult circumstances were often afraid of doing screening and did not want to take examinations when having mild symptoms. (…) However, if the examinations were free, they would go. (interviewee #16, volunteer)

Financial and social support for people screened for and/or detected with TB was another major benefit of ACF that interviewees described, e.g., through the provision of meals for households screened, free services, and financial support to buy food and fuel. A volunteer mentioned providing “moral support” to ACF participants (interviewee #16), while people/patients were appreciative of receiving support, advice, instructions, and care.

He [the employee] gave me advice and comforted me. And that made me happy because I was sick and he made me feel like there was someone who cared for me. (interviewee #33, patient)

### Individual professional

Interviewees from all stakeholder groups highlighted the importance of the dedication and motivation of the employees and volunteers, their experience and skills, and good communication. Furthermore, volunteers emphasized that it was crucial to have a network to facilitate ACF implementation. These four categories are further explained in the following.

Interviewees often described the dedication and motivation of employees and volunteers, being expressed by providing support, care, and encouragement to people/patients and by showing passion and enthusiasm. Negative attitudes described were mostly about the lack of enthusiasm. Examples of how employees and volunteers showed dedication and motivation included: adjusting their schedule to match availability of participants, driving people to the testing facility, conducting follow-ups of patients, and providing them with their contact details. A District TB Unit leader elaborated on the “spirit” of the volunteers:

Others might work for money, but the volunteers would work because of their volunteer spirit. But they would work voluntarily only when they were focused and understood what they could do for the community. (interviewee #19, leader)

This “spirit” was also reflected on by employees and volunteers, describing ACF as meaningful and beneficial, and mentioning that people in the community thanked them for their work. Patients expressed their gratitude toward employees and volunteers, e.g., saying they “explained and encouraged [them] wholeheartedly” (interviewee #22).

Employees and volunteers had experience and skills useful for ACF implementation. IMPACT TB also provided them with opportunities to share experiences to overcome implementation challenges. Skills said to be crucial were related to communication, persuasion, advising and approaching people, making people feel comfortable, and developing trust. Some employees and volunteers had previously worked in other local community-based projects, disease areas, public affairs, or in collaboration with the health facility. At the same time, a District TB Unit leader cautioned that experienced employees and volunteers who were still involved in various projects outside of IMPACT TB were prone to being overburdened.

*They [people who had participated in social work] were enthusiastic, but they had too many responsibilities. In the end, they couldn’t handle the job because of pressure from their area. (interviewee #39, leader)*

Volunteers explained how having a network and local relationships could help ACF implementation; such networks and relationships were built over the course of IMPACT TB or were already in place, e.g., membership of local associations, resident groups, or networks based on previous work experience. This was the only category mentioned by volunteers and not by employees.

*We often worked as collaborators, volunteers and we were often housewives, group leaders or from the Women’s Union, Association of the Elderly, we knew the area to conduct activities well. The new ones to the area would find it difficult to approach people. (interviewee #16, volunteer)*

Good communication was among the key factors facilitating ACF implementation, meaning interpersonal communication between employees and volunteers and TB patients; their families and the community to establish trust, resolve conflicts, or persuade people to participate in ACF; and interpersonal communication among community members (e.g., “word of mouth”).

*I think we need to improve the ability to convince people. We should find consultants who are good at convincing others. I think that’s what matters. […] Everyone will listen to you if you’re persuasive enough. (interviewee #31, employee)*

Moreover, a leader explained that employees and volunteers who understand the local context and speak the local dialect may be more successful in persuading people to participate in ACF:

*The staff from [the hospital] can’t be as close to the people as the volunteers who are recruited there. Because they will understand – not to mention the difference in dialect and tone. (…) Having collaborators speaking the regional dialect will increase persuasion. (interviewee #8, leader)*

To facilitate communication, employees and volunteers used tools such as flyers, platforms such as meetings of residential groups, and communication channels such as the social networking app *Zalo*. A leader stressed the need for a dedicated budget for communication and advocacy.

### Patient

Interviewees from all stakeholder groups emphasized limited participation from ACF participants and TB patients, the prevalence of stigma, discrimination and fear, and trust and mistrust as factors influencing ACF implementation, which we grouped into three categories.

TB-related stigma, discrimination, and fear were described as barriers for ACF implementation. People were afraid of the disease itself, of others staying away from them, or of losing their work or home. Therefore, they would refuse participation in ACF or hide their disease. In addition, the health and safety of the employees and volunteers were said to be impacted upon by stigma, e.g., a volunteer did not wear a face mask to avoid alienating the person screened. However, other forms of airborne infection prevention and control were used, including implementation that took place outdoors (i.e., in well-ventilated areas).

*I even didn’t dare to wear a face mask. Even though we knew that they had TB, we had to accept the fact that we couldn’t wear face masks and explained to assure them that they were not a burden. (interviewee #20, volunteer)*

To prevent TB-related stigma, discrimination, and fear, a staff member of the National TB Programme stated that ACF would often be announced as “lung health screening” rather than “TB screening.” Patients suggested that it would be preferable to be contacted via telephone, rather than personal visits to learn about ACF, and for health workers to distribute invitations for ACF not only to selected groups, but to whole communities to avoid discrimination. A volunteer made similar suggestions.

Limited participation was linked to stigma, discrimination, and fear. Moreover, misperceptions (e.g., that ACF was being implemented to sell medicines), disbelief (e.g., of having TB), not wanting to know their disease status, or being too busy were believed to lead to limited participation. Having health insurance was reported to limit some people’s willingness to participate in ACF, as they thought the insurance would offer protection in case of disease:

*I encouraged the patients’ family members to take the CXR screening test, but they refused. They said they didn’t have time, or they already had their health insurance. (interviewee #21, volunteer)*.

Mistrust among ACF participants and local public health authorities was another reason limiting participation. A patient stated that “sometimes people won’t believe in these free things” (interviewee #32), which was also reflected in a similar statement made by an employee. Employees, volunteers and patients described how trust developed over time: I usually don’t believe in other’s words, but [the employee] is different. She said if I didn’t believe, she would drive me there, and gave me some money to go home. […] Then she drove me to the exam address. After finishing the exam, that’s when I began to trust her. (interviewee #32, patient).

Implementer’s knowledge, behavior, attitude, and appearance were described as important to gain trust from people, e.g., volunteers and employees should communicate clearly and truthfully, be friendly, and kind. A patient described how the appearance of the implementer may impair trust, saying “her bicycle is too old; people may think she’s a fraud,” and suggested for her to be provided with a vehicle (interviewee #32).

### Social context

Different stakeholders’ collaboration and engagement, commitment and support, and the need to improve communication and awareness-raising were emphasized by interviewees from all stakeholder groups as important facilitators for ACF and grouped into three categories.

“We cannot do this [ACF] alone”, said an employee (interviewee #25). Many interviewees highlighted good collaboration with and engagement of different stakeholders, and their commitment and support as crucial facilitators for ACF implementation. Stakeholders included the local government, the city’s Public Health Association, District TB Units, health stations and staff in charge of TB, unions, People’s Committees of the districts and wards (i.e., local government), community leaders, residential groups, project coordinators, employees and volunteers. A leader stressed the importance of a common understanding of ACF among all stakeholders involved. Relations with local authorities and between colleagues were emphasized:

*We should assign one or two leaders to manage each area. The first reason is that they have relationships with local government. The second reason is that they have relationships with their colleagues. And the final reason is that they can easily organize, direct or implement activities. (interviewee #39, leader)*

The need to improve communication and awareness-raising at all levels was pointed out by the interviewees to help prevent TB and/or increase participation in ACF. Different communication channels, such as television, radio, and social media, were talked about. Interestingly, a leader stressed that reading materials had limited effectiveness and that communication efforts should rather focus on direct communication to the community. Patients suggested that employees and volunteers visit individual households or make announcements in the community to invite people to participate in ACF.

### Organizational context

Interviewees from all stakeholder groups elaborated on the availability of and need to retain human and financial resources, level of incentives for employees and volunteers, and challenges with preparation and logistics, as factors influencing ACF. Employees, volunteers, and leaders commented on the importance of capacity-building as part of ACF. We grouped these factors into four categories.

Interviewees highlighted that ACF implementation required additional human and financial resources; they acknowledged IMPACT TB, firstly, for providing funds for human resources and capacity-building, leading to a “longer, sustainable impact” (interviewee #4, leader) and, secondly, for offering “favorable conditions […] such as funds for screening tests [CXR], travel, sputum transportation, etc.” (interviewee #34, leader).

Employees and volunteers appreciated monetary and non-monetary incentives, e.g., the opportunity for capacity-building, which increased their confidence and empowered them to implement ACF. However, incentives were often said to be insufficient or lacking.

*In terms of supporting money from the program for the collaborators, if possible, the program should raise a little so that each collaborator could cover some of their expenses. This is my suggestion, not demand. (interviewee #16, volunteer)*

Challenges with preparation and logistics were another barrier for ACF implementation. Employees and volunteers described the difficulty of contacting, arranging times with, and locating prospective ACF participants, e.g., as people would be working, lived in faraway districts or because their addresses were unavailable, out of date, or false. Locating people was particularly difficult in the case of internal migrants and other mobile populations. Employees tried to mitigate such challenges by collaborating closely with the District TB Units and public health authorities.

An NTP staff member emphasized that preparation was especially important in the beginning of the ACF project, considering the learning curve of an implementer in putting ACF into practice. Suggestions for improvement included making a detailed plan before implementation, having comprehensive instructions for the employees and volunteers, assessing the results of the ACF projects and using those to improve future activities. Such plans and instructions existed, but it is unclear if the interviewees were aware of them.

*The coordinators should be provided with detailed instructions of what and how they will do, or the amount they will receive. Everything must be as clear as possible so that they could work enthusiastically and dedicatedly. Unclear information or instructions may cause inefficiency. (interviewee #19, leader)*

## Discussion

Our qualitative study explored the perceptions of key stakeholders regarding the facilitators and barriers for ACF implementation in six districts of HCMC. We generated three main themes from the data: (1) the studied ACF model provided a conducive social and organizational context for ACF implementation with areas for improvement, including communication and awareness-raising, preparation and logistics, data systems and processes, and incentives. (2) Employees and volunteers capitalized on their strengths to facilitate ACF implementation, e.g., experience, skills, and communication. (3) Employees and volunteers were in the position to address patient-level barriers to ACF implementation, e.g., stigma, discrimination, and mistrust. All categories were mentioned by employees and volunteers, except the category of having a network that facilitates ACF implementation, which was only mentioned by volunteers. The themes covered a variety of facilitators and barriers, which we divided into 17 categories.

Our study confirms that virtually all identified facilitators and barriers for this model of ACF implementation are in line with the existing evidence, as outlined in a recent scoping review on the topic [19]. While many studies examining facilitators and barriers for ACF implementation focus on descriptions of what these factors are, our study also provided examples and ideas of how to address them. To name a few examples of how our results are aligned with the literature, our results show that employees and volunteers’ experience, skills, and motivation influence ACF implementation, which other studies have also described [20–22, 31, 32]. For instance, a qualitative study conducted in Kampala, Uganda, described the provision of personalized care and enabling TB patients support as a key facilitator for TB contact investigation [20]. Moreover, in line with our findings, many studies pinpoint stigma, discrimination, and fear as barriers for ACF [33–39]. This study also confirms the importance of collaboration and engagement of key actors, such as District TB Units, health stations, and employees and volunteers, as has also been found in settings such as Uganda [20], Cambodia [38], Thailand, and Myanmar [40]. While it is difficult to weigh facilitators and barriers against one another, our study showed that, apart from overcoming barriers, capitalizing on facilitators was important to achieve successful implementation. Future implementation strategies should address both facilitators and barriers at all levels.

In terms of progressing beyond describing what the facilitators and barriers are, our data also provide examples and ideas of how to address them. Studies from Nigeria [41] and India [42] are among the few studies that also elaborate on the “how-to” of ACF implementation: Shamenewadi and colleagues [42] mentioned involving local leaders, training employees, and volunteers in counseling TB patients and supporting patients financially for ACF implementation. Based on our results, “how-to” examples and ideas included close collaboration between employees and volunteers and staff from District TB Units to overcome challenges with preparation and logistics for ACF in the community. Volunteers mentioned the use of their networks and relationships to easily navigate the districts and to approach people with presumptive TB. Given that the use of networks was the sole factor mentioned by volunteers only, it may have contributed to the lower Number Needed to Screen to detect one case of TB in the volunteer districts, which had been found by Vo and colleagues [23]. Yet, larger changes in TB treatment rates were documented in the employee districts compared to the volunteer districts [23]. The latter study also suggested that the volunteer model could be appropriate in settings where the workload and the opportunity cost of volunteering are low [23]. While these “how-to” examples include fundamental concepts, concrete locally adapted steps should be conceived in detail, e.g., how to foster close collaboration and how to support employees and volunteers that may not have local networks. Such insights on the “how-to” aspects of implementing ACF seem particularly constructive and valuable to inform stakeholders (including researchers, decision-makers, practitioners, and donors) for future ACF implementation and scale-up in Vietnam, and potentially in other high TB burden countries. Indeed, the similarity of our results compared with existing evidence indicates that the identified facilitators, barriers, and “how-to” strategies for ACF could inform ACF implementation across contexts. Future research could provide an in-depth understanding of those influencing factors and their magnitude in specific contexts.

Finally, our results stressed the importance of a people-centric approach to ACF; TB patients were placed at the center of ACF implementation, as many categories illustrated (e.g., focusing on access, patient support, and trust). This is also in line with WHO guidance [3]. In addition, ACF employees and volunteers were at the center of ACF implementation. They “make or break” ACF implementation, being frequently vulnerable populations themselves, who require support. Employees and volunteers, or community health workers more broadly, are often poor and dependent on project-based salaries and/or incentives [43]. We suggest that increased capacity, knowledge, and empowerment of employees and volunteers that accompanies ACF implementation should be considered as positive factors for ACF. At the same time, employees and volunteers should become an integral part of the health system while efforts should be made to make the conducive social and organizational context in which they work sustainable and independent of project funding. Shifting mindsets by putting employees and volunteers at the center of ACF implementation, alongside TB patients, may lead to more effective and durable ACF implementation.

### Strengths and limitations

A strength of this study is the inclusion of different stakeholder groups involved in IMPACT TB ACF implementation. Recruited across multiple levels of the health system, they provided diverse insights into the topic. Moreover, the collaboration with experienced data collectors who were familiar with the Vietnamese context, including ACF and the Vietnamese health system, helped to generate high-quality, contextualized data for our study. In addition, the triangulation of data and extensive discussion among members of the research team enabled us to better understand and ascertain the dependability, accuracy, breadth, and depth of the data collected. This study adds value by providing concrete insights into how to strengthen facilitators and how to overcome barriers. While MC and KL had been involved in the planning of IMPACT TB, RJF, LNQV, and AJC had been closely involved in planning and/or implementation. OB, PBT, and KV did not have any role in planning or implementing IMPACT TB. While the insider perspective of some members of the research team strengthens the current study by including both outsider and insider perspectives on data collection and analysis, it may have also introduced bias.

In terms of limitations of this study, some selection and social desirability bias was likely present among the interviewees. The programmatic data we reviewed revealed that the employees and volunteers who participated in the study were among the highest performing health workers in terms of project outputs. It may be possible that high-performing implementers faced the same barriers as low-performing ones but that they were better able to overcome them. Yet, we acknowledge that low-performing implementers may have had different views on ACF and hope future research may address this gap. Social desirability bias may have also been present among the interviewees. However, the data collectors tried to mitigate this bias by building rapport with the interviewees and by following up statements that were made by interviewees with clarifying and probing questions during the interviews rather than strictly adhering to the interview guides. Furthermore, some interviewees, especially leaders, occasionally conflated IMPACT TB ACF implementation with the broad topic of ACF/TB control. In these events, interviewees were directed to discuss their specific experiences. In addition, interviewees’ rationale for implementing/participating in ACF was not always clear, which may affect the reliability of the data.

## Conclusion

This study provides new insights into facilitators, barriers and "how-to" strategies for the implementation of ACF in Vietnam, as perceived by key stakeholders. The IMPACT TB project provided a favorable social and organizational context for ACF implementation. Yet, individual employees and volunteers still determined the success of the process, as they had to be able to capitalize on their own strengths while also addressing patient-level barriers. Volunteers especially used their networks to facilitate ACF. Knowledge of both facilitators and barriers, and how to address them can inform the planning and implementation ACF in Vietnam and similar contexts across low- and middle-income countries worldwide.

## Supplementary Information

**Additional file 1.** COnsolidated criteria for REporting Qualitative research (COREQ) checklist

**Additional file 2.** Interview guides

## Data Availability

The datasets generated and analyzed during the current study are not publicly available. The informed consent that all interviewees signed promised full anonymity. Following data requests, interview transcripts will be reviewed for any potential identifying information and will only be made available to researchers who sign a data sharing agreement. Data requests may be sent to the corresponding author.

## Abbreviations

ACF: Active case-finding
COREQ: Consolidated criteria for reporting qualitative research
CXR: Chest X-ray
HCMC: Ho Chi Minh City
IMPACT TB: Implementing proven community-based active TB case finding intervention
NTP: National TB Programme
PCF: Passive case-finding
TB: Tuberculosis
WHO: World Health Organization
