# Supplementary material for: Capitalizing on facilitators and addressing barriers when implementing active tuberculosis case-finding in six districts of Ho Chi Minh City, Vietnam: a qualitative study with key stakeholders
Source: Implement Sci. 2021 May 19;16:54. doi: 10.1186/s13012-021-01124-0 (PMC8135167; doi:10.1186/s13012-021-01124-0)
Supplement: Supplementary file 2 — Additional file 2. Interview guides [file 13012_2021_1124_MOESM2_ESM.docx]

**Additional file 2 – Interview guides**

**Stakeholder group 1: Employees and volunteers**

1. Could you please describe your experience in implementing IMPACT TB’s ACF?

Thinking about IMPACT TB’s ACF and the way that it was put into practice:

1. What are three things you liked most about the implementation?
   - Why?
2. What were the three biggest challenges you faced in the implementation?
   - Were you able to overcome these challenges?
     - How?
   - Have any patients ever refused to be screened when you approached them? What were the reasons for refusal?
3. From your perspective, how could ACF be done better in the future?
4. What could *others* (e.g. other employees/volunteers) do to better implement ACF?
5. If you could do it again, what would *you* do to better implement ACF?
   - Do have experience with implementing other ACF projects?
     - If yes, what was the difference to IMPACT TB’s ACF implementation?
6. Do you have any additional comments?

**Stakeholder group 2: Patients**

1. Could you please describe your experience of when you met the volunteer/employee who screened you for TB?

Thinking about this TB screening activity and the way it was done:

1. What are three things you liked most about the screening?
   - Could you please elaborate on why you liked these?
2. What were your three biggest challenges in participating in the screening?
   - How did you overcome these challenges?
   - Do you know of other TB patients like yourself that are supported/advised by the volunteers/employees? Do they face any challenges?
3. Have you ever heard of any patients that refused to be screened when the volunteer/employee approached them? What were the reasons for refusal?
4. How do you think the screening could be done better for other TB patients in the future?
5. Do you have any additional comments?

**Stakeholder group 3: Leaders from district, national or international institutions and organizations in Vietnam**

Part 1

1. Could you please describe your experience with ACF implementation?

Thinking about ACF implementation:

1. Which do you consider the three most important facilitators for ACF implementation?
   - Why?
2. Which are the three most important barriers for ACF implementation?
   - Why?
3. From your perspective, how could ACF be done better in the future?
4. What could *others* (e.g. other employees/volunteers) do to better implement ACF?
5. What could *you* do to support better implementation of ACF?

Part 2 (Only use if person has experience with ACF data.)

1. Please describe your experience in working with ACF data.
2. From your experience, which are the three most important factors influencing the quality of ACF data in Vietnam?
   - At which stage do these factors influence the data quality, i.e. data collection, data entry, data analysis or any other?
3. How could the quality of ACF data in Vietnam be improved in the future?
4. Looking at the notifications in the IMPACT TB implementation and control districts (*Show the interviewee two graphs based on FIT’s trend analyses*)…
   - How do you see ACF implementation barriers and facilitators reflected in those data/trends?
5. What lessons do the data from IMPACT TB teach us about ACF implementation?
6. How could the data from IMPACT TB be useful for informing ACF policy and practice in Vietnam?
   - What other evidence on ACF implementation (e.g. from operational studies) could/does inform policy and practice about ACF in Vietnam?
7. Do you have any additional comments?
